# Rates and impact of mental disorders among Australian youth: prevalence, severity, and trends from two national psychiatric surveys

**DOI:** 10.1016/j.lanwpc.2026.101925

**Published:** 2026-07-23

**Authors:** Louise Birrell, Tim Slade, Sam Jones, Matthew Sunderland, Lucinda Grummitt, Lexine Stapinski, Katrina Prior, Georgie Berry, Ivana Kihas, Nicola C. Newton, Julia Macauley, Maree Teesson, Cath Chapman

**Affiliations:** a The Matilda Centre for Research in Mental Health and Substance Use, The University of Sydney, NSW, 2006, Australia

**Keywords:** Youth, Mental health, Anxiety disorders, Mood disorders, Substance use, Epidemiology, Prevalence

## Abstract

**Background:**

Mental disorders are leading contributors to disease burden among young people. Recent evidence suggests rising rates of these disorders, yet no recent national comparisons using diagnostic interviews have examined changes in individual disorder prevalence among Australian youth over time. This study provides current estimates of prevalence, patterns, severity, and profiles of mental disorders among Australians aged 16–24, comparing data from 2007 to 2020–2022.

**Methods:**

Data were drawn from the 2020–2022 National Study of Mental Health and Wellbeing (NSMHWB) and compared with the 2007 NSMHWB. A nationally representative sample of 16–24-year-olds completed the WMH-CIDI to assess 12-month DSM-IV mood, anxiety, and substance use disorders, along with measures of severity, comorbidity, suicidality and disability. Analyses were population-weighted and accounted for complex survey design. Temporal changes were examined using logistic and proportional odds regression.

**Findings:**

The 12-month prevalence of mental disorders increased from 23.4% in 2007 to 36.2% in 2020–2022 (OR 1.86, 95%CI 1.53–2.25). Mental disorders continue to be more common in females, but increases were also observed for males. Anxiety disorders nearly tripled (12.2% to 29.1%; OR 2.94, 95% CI 2.33–3.71), and mood disorders rose from 4.9% to 13.3% (OR 3.01, 95% CI 2.16–4.20), while substance use disorders declined (12.34% to 7.76%; OR 0.60, 95% CI 0.44–0.80). Comorbidity, severity, suicidal thoughts, psychological distress, and disability also rose, with one in five experiencing multiple disorders in 2020–2022.

**Interpretation:**

Mental disorders among Australian youth have increased markedly since 2007, with greater severity and functional impairment.

**Funding:**

The Australian National Health and Medical Research Council.

**Research in context**

**Evidence before this study**

Accurate population prevalence rates for youth mental health and substance use disorders are key estimates to inform national policy, intervention and prevention efforts. Examining change over time in population prevalence among young people in Australian provides key insights into changing trends at a national level. Previous population prevalence studies using diagnostic criteria typically haven’t explored prevalence and change over time specifically for young people or at the individual disorder level. We searched PubMed and Scopus on the 9th October 2025 with the following search terms: ((“mental disorder∗”[tiab] OR “anxiety disorder∗”[tiab] OR “mood disorder∗”[tiab] OR “substance use disorder∗”[tiab]) AND (prevalence[ti] OR epidemiolog∗[ti]) AND (“young adult∗”[tiab] OR youth[tiab] OR “young people”[tiab] OR “16–24”[tiab] OR “16 to 24”[tiab])) AND (“population-based”[tiab] OR “national survey”[tiab] OR “household survey”[tiab] OR “general population”[tiab] OR “National Study of Mental Health and Wellbeing”[tiab] OR “National Survey of Mental Health and Wellbeing”[tiab] OR NSMHWB[tiab] OR NSMHW[tiab]), for articles in any language published in the past 10 years. After removing duplicates, we identified 104 articles, including 5 systematic reviews. We also searched for relevant articles reporting youth prevalence rates in the past 10 years (publication date >2014) from the World Mental Health Survey Initiative publication site (https://www.hcp.med.harvard.edu/wmh/publications.php). Most studies reported prevalence estimates across all age groups for mental disorders in one country, with one book chapter providing a summary of estimates from the World Mental Health Survey Initiative across multiple countries and regions. While many national prevalence studies examined the impact of age on likelihood of experiencing a mental disorder this was typically expressed as an odds ratio, with prevalence rates not disaggregated by age groups. Few studies examined change in national prevalence over time, and none examined change over time in youth prevalence specifically or by individual type of disorder.

**Added value of this study**

Using data from the 2007 and 2020–2022 National Survey of Mental Health and Wellbeing, we found a significant increase in the 12-month prevalence for any mental disorder from 23.4% in 2007 to of 36.2% in 2020–2022 among those aged between 16 and 24 years-of-age. Overall, mental disorders continue to be more prevalent among young females, although rates of individual disorders rose for both females and males from 2007 to 2020–2022. Some of the largest increases were seen in individual anxiety disorders, notably social phobia and agoraphobia, while alcohol use disorder declined across this period. Co-morbidity, severity, psychological distress, suicidal ideation and functional impairment (evidenced by greater number of disability days attributable to mental disorders) also substantially increased for young people between 2007 and 2020–2022. While alcohol use disorder declined overall, cannabis and drug use disorders remained stable and for those young people who did experience a substance use disorder it was more likely to be a higher severity disorder.

**Implications of all the available evidence**

Mental disorders are increasingly common and more severe in young people aged 16–24 years-of-age. The updated estimates highlight the growing impact of mental disorders at a population level and highlight key targets for intervention, including young females and anxiety disorders.

## Introduction

Mental disorders typically first emerge across childhood, adolescence to young adulthood,^1^ with, 63% of lifetime cases onsetting before age 25.^2^ As these years represent a critical developmental window for the onset of anxiety, mood and substance use disorders, accurate age-specific epidemiological monitoring is critical. However, national epidemiological data rarely provide youth specific, diagnostic interview-based estimates that can be compared across time, limiting the ability to evaluate real changes in youth population mental health.

Recent data suggest rates of mental health disorders are common among young people and increasing in prevalence in many countries.^3^ In contrast, substance use disorders, particularly alcohol use disorder, have declined in this age group.^4^ However, while overall rates of substance use may have declined, alcohol and cannabis use disorders remain major contributors to morbidity and mortality in adolescents and young adults.^5^ Interpretability of these trends is constrained by variation in measurement methods and inconsistent age groupings across studies. Robust assessment of temporal change requires longitudinal diagnostic comparability, that is, the use of consistent diagnostic instruments, algorithms and sampling frames across survey waves. Without this, it remains unclear whether observed increases reflect true rises in disorder prevalence and burden.

Beyond prevalence alone, severity, functional impairment and comorbidity patterns are critical for understanding the clinical and public health significance of mental disorders in youth. Rising prevalence could reflect an increase in milder presentations, an escalation in complex and impairing disorders, or shifts in the co-occurrence of anxiety, mood and substance-related conditions. Comorbid disorders are typically more severe, harder to treat, and associated with greater service need^6^ therefore, incorporating severity and comorbidity profiling provides essential insight into whether changes over time represent shifts in case complexity rather than simply increases in prevalence.

While mental disorders and substance use disorders (henceforth referred to as mental disorders) can be experienced by anyone, key population subgroups are more likely to experience poor mental health. This includes those assigned female sex at birth,^7^ people who are unemployed,^8^ unmarried,^9^ and living in lower socio-economic status areas.^10^ Identifying youth subgroups at high risk for mental disorders is essential for informing policy and developing prevention strategies.

Australia’s 2020–2022 National Study of Mental Health and Wellbeing (NSMHWB) provides the first opportunity since 2007 to examine youth mental disorders using comparable, structured diagnostic interviews in a nationally representative sample.^11^

This study will provide up-to-date estimates of the prevalence, patterns, profiles and severity of mental disorders amongst a contemporary, nationally representative sample of young people aged 16–24 years of age and examine change over time from 2007 to 2022.

## Methods

### Sample

This study presents findings from 16 to 24-year-olds in the 2020–2022 National Study of Mental Health and Wellbeing (NSMHWB), with the 2007 survey, employing similar sampling and survey design methodology, used to assess changes over time. The complex survey design, sampling framework and administration can be found in Slade and Colleagues.^11^^,^^12^ The 2020–2022 NSMHWB collected data from two cohorts, the first between December 2020 and July 2021 and the second between December 2021 to October 2022. These samples represented all residents in Australia aged between 16 and 85 years living in private dwellings across all states and territories. Very remote locations, including discrete Aboriginal and Torres Strait Islander communities, were not included. The response rates were 60% in 2007 and 52% in 2020–2022. The Confidentialised Unit Record File (CURF) was used to calculate prevalence values for the 2007 survey. The use of data and comparisons between surveys was approved by the Australian Bureau of Statistics (ABS) and conducted within their DataLab, a secure virtual data analysis environment. Results were vetted in accordance with ABS data safety rules before release. As a secondary analysis of publicly available, de-identified data from the ABS, this study was exempt from human research ethics review and individual participant consent, consistent with University of Sydney and the National Statement on Ethical Conduct of Human Research guidelines for low-risk research using existing, non-identifiable data. Approval was obtained from the ABS to access the data for this study.

### Measures

#### Mental disorders

Diagnostic information was assessed using the interviewer-administered version of the World Mental Health Consortium Composite International Diagnostic Interview (WMH-CIDI). This survey has strong psychometric properties and has been used extensively as part of the World Mental Health Survey Initiative. Lifetime individual mental and substance use diagnoses were determined using the criteria prescribed in the Diagnostic and Statistical Manual of Mental Disorders, 4th edition (DSM-IV). Onset and recency questions were used to determine whether each disorder was present in the 12 months prior to interview. The definition of severity described in Slade, Vescovi^11^ was used to determine the severity of mental health disorders.

Mood disorders included major depressive disorder, bipolar disorder and dysthymia. Anxiety disorders included agoraphobia with or without panic disorder, social anxiety disorder, panic disorder, generalised anxiety disorder (GAD), obsessive-compulsive disorder (OCD), and post-traumatic stress disorder (PTSD). Substance use disorders included alcohol, cannabis, sedative, stimulant, and opioid use disorders. DSM-IV diagnoses ‘without hierarchy rules’ were used to capture the true extent of comorbidity. Comorbidity was assessed using combinations of disorder classes: mood, anxiety, and substance use disorders.

#### Suicidal thoughts, suicide plans and suicide attempts

Participants were asked whether they had ever seriously thought about taking their own life (thoughts). They were then asked whether they had ever made a plan to take their own life (plans) and whether they have attempted to take their own life (attempts). If yes, respondents were asked whether this happened in the past 12 months.

#### Correlates

Demographic and clinical correlates of disorders included age, sex assigned at birth, country of birth, marital status, employment status, socio-economic status (Socio-Economic Indexes for Areas [SEIFA]; Index of relative socioeconomic disadvantage quintiles with 1 being most disadvantaged and 5 least disadvantaged), remoteness area (ARIA; Accessibility/Remoteness Index of Australia defined as a measure of relative geographic access to services), psychological distress, disorder disability days and smoking status. Psychological distress was measured using the Kessler psychological distress scale (K10) with previously established cut points at 15, 21, and 29 for low, moderate, high and severe psychological distress, respectively.^13^ Disorder disability days were defined as the mean number of days in the past four weeks that participants were completely unable to work, study or manage day to day activities due to their mental health.

### Statistical analysis

All analyses were conducted using the ‘survey’ package in R Statistical Software version 4.4.1, within the secure ABS DataLab environment. To ensure the sample was representative of the general Australian population, weights were calculated based on the inverse probability of selection. In addition, balanced repeated replicate weights were used to account for the complex survey sampling design when estimating standard errors and confidence intervals. Descriptive tables depict the demographic profile of young people in 2007 and 2020–2022 using weighted means and proportions.

The weighted prevalence of each mental health condition was calculated, stratified by sex at birth and survey year. Minimum sample size requirements, in accordance with ABS data suppression rules, meant that some prevalence estimates are not reported. To examine whether mental disorder prevalence changed over time, logistic regression models were run in which survey year (0 = 2007 and 1 = 2020-2020) was the predictor and mental disorder (0 = no, 1 = yes) was the outcome. To explore whether changes over time were evident among demographic subgroups, logistic regression models were run including an interaction between survey year and each demographic variable separately. For all demographic variables except sex at birth we examined any mental disorder as the outcome. For sex at birth, we ran separate models for each individual mental disorder, mental disorder group and any mental disorder.

The proportion of participants experiencing severe, moderate and mild 12-month mental disorder was estimated. Changes in these proportions over time were determined using proportional odds logistic regressions (pOR). Positive odds ratios from these models indicate a shift towards higher severity groups. Suicidality, number of disorders, K10 psychological distress groups, and mean disorder disability days were summarised using weighted prevalences and means, with associated standard errors. The standard mean difference (SMD) was generated for the latter to assess changes between surveys. Acknowledging the risk of chance findings resulting from multiple testing, when interpreting the results we focus on those associations that are statistically significant at the p < 0.00128 level (0.05/39; as we compare the 2007 and the 2020–2022 data on 39 separate outcome variables).

Youth reflections and insights on the findings can be found in a companion report. This included consultation with The Matilda Centre Youth Advisory Board (YAB)^14^ and report write up leadership from a youth advisory coauthor (GB), which supplement the findings of this paper with lived-experience insights and recommendations.

### Role of the funding source

The funders of the study had no role in study design, data collection, data analysis, data interpretation, or writing of the report.

## Results

### Demographics

As depicted in Table 1, the demographic profile of Australian youth has not changed substantially between 2007 and 2020–2022. The proportion living in urban areas increased from 70.7% (SE = 1.02) to 77.0% (SE = 0.94) and lifetime smoking declined from 21.9% (SE = 1.05) to 8.6% (SE = 0.79).

**Table 1:** Survey respondent sociodemographic characteristics of young people in 2007 and 2020–2022 surveys.

	2020–2022			2007		
	Weighted %	Sample count	Weighted count	Weighted %	Sample count	Weighted count
Mean age	19.90	1619	2,709,340	19.68	1471	2,546,908
Sex at birth^a^						
Male	51.40%	794	1,392,531	51.05%	681	1,300,071
Female	48.60%	825	1,316,809	48.95%	790	1,246,837
Employment						
Employed	71.38%	1222	1,934,865	72.26%	1076	1,839,409
Unemployed	7.52%	96	203,733	5.33%	79	135,576
Not in labour force	21.11%	301	572,240	22.41%	316	570,427
Residence						
Metro	77.06%	1314	2,088,975	70.71%	1000	1,799,931
Inner regional	15.50%	205	420,195	18.89%	295	480,734
Outer regional	7.44%	100	201,668	10.40%	176	264,747
Birth country						
Australia	81.76%	1280	2,216,273	83.17%	1224	2,116,902
English speaking	5.19%	93	140,825	4.69%	76	119,437
Not English speaking	13.05%	246	353,740	12.14%	171	309,072
IRSD						
Q1–Q2	31.57%	524	855,726	35.00%	544	890,787
Q3–Q4	44.63%	712	1,209,734	41.06%	605	1,045,131
Q5	23.81%	383	645,378	23.94%	322	609,493

SE: Standard Error; IRSD: Index of Relative Socio-economic Advantage and Disadvantage; Q1 represents highest advantage quartile.

a Total excludes persons who described their sex at birth as another term due to small cell counts and data release restrictions (Australian Bureau of Statistics. (2021, November 19). *DataLab Clearance*. ABS. https://www.abs.gov.au/statistics/microdata-tablebuilder/datalab/datalab-clearance).

### Prevalence

As shown in Tables 2 and 3, past 12-month experience of any mental disorder among 16–24-year-olds significantly increased from one in four in 2007 to one in three in 2020–2022 (23.4% vs. 36.2%; OR 1.86, 95% CI 1.53–2.25). Prevalence in males significantly increased from 20.7% to 30.0% (OR 1.64, 95% CI 1.23–2.19) and females from 26.2% to 42.6% (OR 2.08, 95% CI 1.57–2.76). The prevalence of any mental disorder among demographic subgroups defined by employment status, urbanicity, birth country, or socioeconomic status subgroups has not changed between the 2007 and 2020/22 surveys, see Supplementary Tables S1 and S2.

**Table 2:** Prevalence of 12-month DSM-IV mental disorders among young people in 2020–2022 and 2007 by sex.

	2020–2022						2007					
	Total %	SE	Males	SE	Females	SE	Total %	SE	Males	SE	Females	SE
Major depressive	10.84%	0.82	7.65%	1.11	14.23%	1.29	3.51%	0.50	1.71%	0.54	5.39%	0.86
Dysthymia	1.88%	0.38	1.51%	0.47	2.28%	0.64	NA	NA	NA	NA	NA	NA
Bipolar	2.20%	0.40	1.31%	0.39	3.14%	0.67	1.28%	0.41	NA	NA	NA	NA
Any mood	13.30%	0.79	9.31%	1.10	17.56%	1.31	4.85%	0.70	2.58%	0.72	7.21%	1.03
GAD	5.57%	0.55	3.68%	0.73	7.35%	0.86	1.37%	0.29	NA	NA	NA	NA
Panic	5.94%	0.70	3.03%	0.62	9.02%	1.15	1.64%	0.35	NA	NA	NA	NA
Agoraphobia	5.08%	0.63	1.87%	0.57	8.49%	1.12	0.62%	0.20	NA	NA	NA	NA
Agora w/o panic	3.43%	0.51	1.50%	0.55	5.48%	0.91	0.50%	0.19	NA	NA	NA	NA
Social	17.95%	1.09	12.57%	1.24	23.67%	1.66	4.32%	0.54	2.80%	0.66	5.91%	0.79
OCD	8.08%	0.65	6.40%	0.99	9.65%	1.12	4.20%	0.77	2.94%	0.89	5.51%	1.22
PTSD	6.64%	0.83	4.09%	0.88	9.36%	1.35	3.96%	0.59	1.60%	0.58	6.43%	0.96
Any anxiety	29.07%	1.26	20.99%	1.63	37.45%	1.81	12.23%	1.08	7.08%	1.07	17.60%	1.86
Alcohol	5.51%	0.71	6.76%	1.24	4.20%	0.75	11.05%	0.94	13.10%	1.58	8.92%	1.40
Cannabis	2.51%	0.52	3.44%	0.95	1.54%	0.45	2.44%	0.42	3.41%	0.67	1.43%	0.45
Drug use^a^	2.95%	0.54	4.05%	0.97	1.80%	0.50	3.40%	0.48	4.39%	0.72	2.37%	0.62
Any substance	7.76%	0.85	9.91%	1.49	5.50%	0.84	12.34%	0.97	14.74%	1.65	9.83%	1.43
Any mental disorder	36.20%	1.40	30.04%	1.89	42.58%	1.91	23.41%	1.36	20.70%	1.87	26.24%	2.29

SE: Standard error; GAD: Generalised Anxiety Disorder; OCD: Obsessive Compulsive Disorder; PTSD: Post Traumatic Stress Disorder; NA: Not available due to the underlying cell count comprising of less than 10 people restrictions (Australian Bureau of Statistics (2021, November 19). DataLab Clearance. ABS. https://www.abs.gov.au/statistics/microdata-tablebuilder/datalab/datalab-clearance).

a Drug use disorders included DSM-IV dependence or abuse on one or more of four separate drug classes—cannabis, sedatives, stimulants and opioids.

**Table 3:** Changes over time in the prevalence of DSM-IV mental disorders.

	Total Sample	Males	Females
	OR (95% CI)	OR (95% CI)	OR (95% CI)
Major depressive	3.34^b^ (2.38–4.69)	4.76^b^ (2.27–10.00)	2.91^b^ (1.96–4.32)
Dysthymia	NA	NA	NA
Bipolar	1.73 (0.78–3.84)	NA	NA
Any mood disorder	3.01^b^ (2.16–4.20)	3.88^b^ (2.02–7.46)	2.74^b^ (1.92–3.91)
GAD	4.23^b^ (2.60–6.89)	NA	NA
Panic disorder	3.79^b^ (2.28–6.30)	NA	NA
Agoraphobia	8.55^b^ (4.05–18.05)	NA	NA
Agoraphobia w/o panic	7.08^b^ (2.87–17.48)	NA	NA
Social phobia	4.84^b^ (3.60–6.51)	5.00^b^ (2.89–8.65)	4.93^b^ (3.52–6.91)
OCD	2.01^a^ (1.31–3.07)	2.26^a^ (1.07–4.75)	1.83^a^ (1.06–3.17)
PTSD	1.72^a^ (1.14–2.61)	2.63^a^ (1.05–6.59)	1.50 (0.95–2.37)
Any anxiety disorder	2.94^b^ (2.33–3.71)	3.49^b^ (2.38–5.10)	2.80^b^ (2.08–3.78)
Alcohol use disorder	0.47^b^ (0.34–0.65)	0.48^a^ (0.30–0.78)	0.45^a^ (0.27–0.74)
Cannabis use disorder	1.03 (0.59–1.80)	1.01 (0.49–2.07)	1.07 (0.44–2.65)
Drug use disorder	0.86 (0.54–1.39)	0.92 (0.49–1.70)	0.76 (0.34–1.68)
Any substance use disorder	0.60^b^ (0.44–0.80)	0.64^a^ (0.42–0.97)	0.53^a^ (0.34–0.84)
Any mental disorder	1.86^b^ (1.53–2.25)	1.64^b^ (1.23–2.19)	2.08^b^ (1.57–2.76)

OR: Odds ratio; 95% CI: 95% confidence interval; GAD: Generalised Anxiety Disorder; OCD: Obsessive Compulsive Disorder; PTSD: Post Traumatic Stress Disorder; NA: Not available due to the underlying cell count comprising of less than 10 people (restrictions Australian Bureau of Statistics (2021, November 19)). DataLab Clearance. ABS. https://www.abs.gov.au/statistics/microdata-tablebuilder/datalab/datalab-clearance).

a p-value < 0.05.

b p-value < 0.00128.

The prevalence of past 12-month mood disorders increased significantly from one in every 20 young people to over one in 10 (4.9% vs. 13.3%; OR 3.01, 95% CI 2.16–4.20), with major depressive disorder increasing from 3.5% to 10.9% (OR 3.34, 95% CI 2.38–4.69).

The 12-month prevalence of anxiety disorder nearly tripled, increasing from 12.2% in 2007 to 29.1% in 2020–2022 (OR 2.94, 95% CI 2.33–3.71). Increasing prevalence was observed across most anxiety disorders particularly for agoraphobia (0.6% vs. 5.1%; OR 8.55, 95% CI 4.05–18.05), social phobia (4.3% vs. 17.9%; OR 4.8, 95% CI 3.60–6.51) and generalised anxiety disorder (1.4% vs. 5.6%; OR 4.23, 95% CI 2.60–6.89). Increases occurred in both sexes: among males, anxiety disorder prevalence rose from 7.1% to 21.0% (OR 3.49, 95% CI 2.38–5.10), and among females from 17.6% to 37.5% (OR 2.80, 95% CI 2.08–3.78).

Substance use disorders were the only category to show a significant decline between 2007 and 2020–2022 (12.3% vs. 7.8%; OR 0.60, 95% CI 0.44–0.80), driven primarily by significant reductions in alcohol use disorder (11.1% vs. 5.5%; OR 0.47, 95% CI 0.34–0.65). Cannabis use disorder remained stable (2.4% vs. 2.5%; OR 1.03, 95% CI 0.59–1.80), as did drug use disorder (3.4% vs. 2.9%; OR 0.86, 95% CI 0.54–1.39).

### Severity

As shown in Table 4, the proportion of Australian youth with a mental disorder in the past year classed as severe increased significantly from 14.7% to 24.0% (SE = 2.1 and 2.4) between the two surveys. Changes in severity between the 2007 and 2020/22 surveys are shown in Table 5. Overall, young people in 2020–2022 were 2.43 times (95% CI 1.88–3.15) more likely to fall into a higher severity category than in 2007. This increase was evident for substance use disorders (pOR 2.27, 95% CI 1.46–3.57). While descriptively, as shown in Table 4, the percentage of mood and anxiety disorder classed as severe increased from 2007 to 2020–2022, according to the stringent p-value threshold there was no statistically significant severity change (pOR 1.57, 1.13–2.18; pOR 1.24, 0.75–2.04). Among females, the likelihood of being in a more severe category was 3.4 times higher in 2020–2022 compared with 2007 (95% CI 1.62–7.18), and among males, 1.8 times higher (95% CI 1.01–3.18).

**Table 4:** Severity of 12-month DSM-IV mental disorders in 2020–2022 and 2007 by sex.

	2020–2022						2007					
	Total %	SE	Males	SE	Females	SE	Total %	SE	Males	SE	Females	SE
Any mood disorder												
Severe	46.09%	4.22%	41.95%	6.22%	48.41%	5.30%	44.34%	7.54%	45.33%	16.46%	43.96%	7.06%
Moderate	48.13%	4.28%	NA	NA	NA	NA	NA	NA	NA	NA	NA	NA
Mild	5.78%	1.75%	NA	NA	NA	NA	NA	NA	NA	NA	NA	NA
Any anxiety disorder												
Severe	26.63%	2.14%	22.06%	3.26%	28.88%	3.31%	20.48%	3.33%	19.05%	5.40%	21.09%	3.98%
Moderate	57.92%	2.40%	56.99%	4.26%	58.83%	3.50%	51.00%	3.95%	47.85%	10.01%	52.31%	3.76%
Mild	15.45%	2.08%	20.94%	3.85%	12.29%	2.09%	28.52%	4.28%	33.10%	9.94%	26.60%	4.34%
Any substance use disorder												
Severe	22.42%	4.62%	12.16%	4.85%	41.99%	8.74%	12.49%	2.73%	7.74%	2.47%	19.90%	6.41%
Moderate	38.98%	6.26%	41.24%	8.36%	NA	NA	32.82%	4.58%	34.03%	5.35%	30.92%	8.42%
Mild	38.60%	6.44%	46.60%	8.00%	NA	NA	54.69%	4.49%	58.22%	5.23%	49.17%	8.10%
Any mental disorder												
Severe	23.95%	2.05%	18.38%	2.68%	27.68%	3.03%	14.72%	2.44%	10.64%	3.19%	18.07%	3.24%
Moderate	55.05%	2.45%	52.29%	3.93%	57.41%	3.51%	40.55%	3.05%	36.20%	5.06%	44.12%	3.90%
Mild	21.00%	2.33%	29.32%	3.84%	14.91%	2.41%	44.73%	3.17%	53.15%	5.07%	37.80%	4.36%

SE: Standard error; NA: Not available due to the underlying cell count comprising of less than 10 people restrictions (Australian Bureau of Statistics (2021, November 19). *DataLab Clearance*. ABS. https://www.abs.gov.au/statistics/microdata-tablebuilder/datalab/datalab-clearance).

**Table 5:** Proportional odds logistic regression to identify changes in severity of DSM-IV mental and substance use disorders between 2007 and 2020–2022.

	Overall	Males	Females
	OR (95% CI)	OR (95% CI)	OR (95% CI)
Any mood disorder	1.24 (0.75–2.04)	1.30 (0.48–3.54)	1.23 (0.68–2.2)
Any anxiety disorder	1.57^a^ (1.13–2.18)	1.20 (0.64–2.24)	1.84^a^ (1.24–2.75)
Any substance use disorder	2.27^b^ (1.46–3.57)	1.79^a^ (1.01–3.18)	3.37^a^ (1.62–7.18)
Any mental disorder	2.43^b^ (1.88–3.15)	2.67^a^ (1.79–4.02)	2.28^a^ (1.62–3.22)

OR: Odds ratio; 95% CI: 95% confidence interval.

a p-value < 0.05.

b p-value < 0.00128.

### Impact

As shown in Tables 6 and 7, suicidal thoughts increased significantly between 2007 and 2020–2022, rising from 3.4% to 6.6% (OR 2.02, 95% CI 1.35–3.02), while plans (1.0% vs. 2.3% OR 2.23, 95% CI 1.50–6.61) and attempts remained relatively stable (0.9% vs. 1.0%; OR 0.88, 95% CI 0.41–1.89). In 2020–2022, males were 3.2 times more likely to report suicidal thoughts compared with 2007 (95% CI 1.50–6.61), and females were 1.6 times more likely (95% CI 1.01–2.67).

**Table 6:** Impact and severity of 12-month DSM-IV mental disorders in 2020–2022 and 2007 by sex.

	2020–2022						2007					
	Total %	SE	Males	SE	Females	SE	Total %	SE	Males	SE	Females	SE
Suicidality												
Thoughts	6.55%	0.68	5.10%	0.89	8.10%	1.04	3.35%	0.54	1.68%	0.50	5.10%	0.96
Plans	2.27%	0.40	1.77%	0.52	2.81%	0.62	1.03%	0.34	NA	NA	NA	NA
Attempts	0.94%	0.26	NA	NA	NA	NA	1.06%	0.25	NA	NA	NA	NA
Comorbidity												
No disorder	54.26%	1.37	61.33%	1.75	46.92%	1.99	64.58%	1.31	66.81%	2.12	62.26%	2.01
One disorder	26.27%	1.01	24.48%	1.21	28.23%	1.62	24.78%	0.78	25.82%	0.98	23.69%	1.10
Two disorders	14.50%	0.69	9.58%	0.91	19.49%	0.83	7.20%	0.55	5.57%	0.48	8.90%	0.97
Three or more disorders	4.97%	1.37	4.60%	2.04	5.37%	1.81	3.45%	1.45	1.81%	2.21	5.15%	2.40
Psych distress (K10)												
Low	49.96%	1.34	58.36%	1.94	41.16%	1.79	67.16%	1.39	73.13%	2.02	60.93%	1.97
Moderate	24.22%	1.07	23.73%	1.61	24.79%	1.48	23.55%	1.32	20.78%	1.91	26.44%	1.94
High	17.41%	0.78	13.18%	1.12	21.94%	1.30	6.64%	0.76	4.98%	0.98	8.37%	1.25
Very high	8.41%	0.78	4.73%	0.81	12.11%	1.31	2.66%	0.57	NA	NA	NA	NA
Disorder disability days	Mean	SEM	Mean	SEM	Mean	SEM	Mean	SEM	Mean	SEM	Mean	SEM
Mood	6.64	0.59	4.52	0.68	7.83	0.83	5.58	1.28	9.54	4.52	4.11	0.91
Anxiety	4.8	0.36	2.99	0.40	5.9	0.48	3.1	0.66	2.33	0.63	3.42	0.94
Substance use	4.78	0.99	3.97	1.16	6.32	1.54	1.79	0.27	1.46	0.38	2.26	0.48

SE: Standard error; K10: Kessler psychological distress scale; Disorder disability days: The number of days in the past month where the participant was completely unable to work or carry out normal activities; SEM: Standard error of the mean. NA: Not available due to the underlying cell count comprising of less than 10 people restrictions (Australian Bureau of Statistics (2021, November 19). DataLab Clearance. ABS. https://www.abs.gov.au/statistics/microdata-tablebuilder/datalab/datalab-clearance).

**Table 7:** Changes in impact and severity of mental health and substance use disorders between 2007 and 2020–2022.

	Total Sample	Males	Females
	OR (95% CI)	OR (95% CI)	OR (95% CI)
Suicidality			
Thoughts	2.02^b^ (1.35–3.02)	3.15^a^ (1.50–6.61)	1.64^a^ (1.01–2.67)
Plans	2.23^a^ (1.00–4.94)	NA	NA
Attempts	0.88 (0.41–1.89)	NA	NA
Comorbidity	1.59^b^ (1.39–1.83)	1.33^a^ (1.08–1.64)	1.86^b^ (1.54–2.25)
Psych distress (K10)	2.18^b^ (1.90–2.51)	1.99^b^ (1.61–2.47)	2.50^b^ (2.07–3.02)

Disorder disability days	Days out of role (Mean) (95% CI)	Days out of role (Mean) (95% CI)	Days out of role (Mean) (95% CI)
Mood disorders	1.06 (−1.74 to 3.85)	−5.02 (−14.13 to 4.09)	3.72^a^ (1.28–6.16)
Anxiety disorders	1.70^a^ (0.21–3.18)	0.66 (−0.82 to 2.14)	2.48^a^ (0.38–4.57)
Substance use disorders	2.99^a^ (0.95–5.02)	2.51^a^ (0.10–4.92)	4.06^a^ (0.86–7.27)

OR: Odds ratio; 95% CI: 95% Confidence interval; Comorbidity: Coded as either no disorders, one, two or three or more disorders; Psych distress: The Kessler psychological distress scale.

NA: Not available due to the underlying cell count comprising of less than 10 people restrictions (Australian Bureau of Statistics (2021, November 19). DataLab Clearance. ABS. https://www.abs.gov.au/statistics/microdata-tablebuilder/datalab/datalab-clearance).

a p-value < 0.05.

b p-value < 0.00128.

Comorbidity was significantly more common in the latest survey, with one in five young people experiencing 12-month co-occurring mental disorders (20.5%, SE = 1.03) compared with one in 10 in 2007 (10.7%, SE = 1.00). Females were 1.9 (95% CI 1.54–2.25) times more likely to experience additional disorders, while males were 1.3 times more likely (95% CI 1.08–1.64) in 2020–2022, compared to 2007.

K10 psychological distress scores increased significantly, with the proportion of young people in the high or very high categories rising from 9.2% (SE 1.35) in 2007 to 25.8% (SE 0.78) in 2020–2022. Distress was higher among females, with one in three (34.1%, SE 1.30) in the top two categories compared with nearly one in five males (18.0%, SE 0.96). This upward shift was reflected in proportional odds ratios: 2.0 for males (95% CI 1.61–2.47) and 2.5 for females (95% CI 2.07–3.02). Overall, young people were at least twice as likely to fall into a higher K10 category compared with 2007.

Disorder disability days (defined as are days where the young person was completely unable to work or perform regular activities in the past 30 days), increased somewhat for anxiety (SMD 1.7, 95% CI 0.21–3.18) and substance use disorders (SMD 3.0, 95% 0.95–5.02), and for females with mood disorders (SMD 3.7 95% CI 1.28–6.16). Mood disorders remained the most disabling overall at 6.6 days impacted over the past 30 (SE = 0.59), with anxiety and substance use both impacting an average of 4.8 days (SE = 0.36 and 0.99). Females with mood disorders recorded the highest impairment at 7.8 days (SE = 0.83).

## Discussion

This study presents updated 12-month prevalence rates of mental disorders, their severity and impact among the Australian population aged 16–24 years of age, using data from the 2020–2022 NSMHWB, the largest life course epidemiological survey of mental disorders in the Australian general population. It also presented comparisons to the 2007 NSMHWB. These findings provide the first opportunity in 15 years to interpret population-level changes using comparable diagnostic methodology, allowing clearer attribution of observed differences to genuine temporal shifts. Results indicated mental disorders are increasingly prevalent, severe and impactful among Australian young people at the population level.

Approximately one in three young people met diagnostic criteria for a mental disorder in 2020–2022. Among these, one in three had an anxiety disorder, one in eight a mood disorder, and one in 13 a substance use disorder. The most prevalent individual disorders were social phobia (18%), major depressive disorder (11%), and obsessive-compulsive disorder (8%). Young people assigned female sex at birth had substantially higher rates of anxiety and mood disorders compared with those assigned male sex at birth, whereas substance use disorders were more common among males (10% vs. 6%).

Of those meeting criteria for a mental disorder, the majority (79%) experienced moderate-to-severe symptoms. The predominance of moderate-to-severe presentations indicates that rising prevalence is not driven by an expansion of milder cases, strengthening the interpretation that recent increases represent a meaningful rise in clinically significant disorder burden. Suicidal thoughts were reported by one in 14 young people, with 2.3% reporting suicidal plans and approximately 1% reporting a suicide attempt within the past year. Overall, one in four young people reported high to severe levels of psychological distress. Functional impairment was considerable. On average, young people with a mood disorder were unable to perform usual activities for about 7 days per month, compared with 5 days for those with an anxiety disorder and 5 days for those with a substance use disorder.

### Comparison with the 2007 national survey of mental health and wellbeing

Comparisons with the 2007 survey indicate increasing prevalence and worsening severity, comorbidity, and functional impact across most mental disorders at the population level. The largest relative increases in prevalence were observed for agoraphobia, social anxiety disorder, and generalized anxiety disorder. Although females continued to have higher overall prevalence than males, relative increases were greater for males across several disorders. For example, in 2020–2022, young males had more than four times the odds of major depressive disorder compared with 2007, vs. a threefold increase for females. Similarly, the odds of an anxiety disorder were 3.5 times higher for males and 2.8 times higher for females compared with 2007. In relation to substance use disorders, young people in 2020–2022 were less likely to meet criteria for an alcohol use disorder, while rates of cannabis use disorder remained stable. These temporal changes are comparable because the same diagnostic interview (WHO-CIDI) and similar sampling procedures were used across surveys. Disorders showing the largest proportional increases, particularly anxiety disorders, align with international evidence of rising internalising symptoms among youth.^15^ The greater relative increases among males may reflect reduced stigma, increased reporting, or genuine deterioration in male mental health. However, future longitudinal research is needed to explore and test underlying causal mechanisms.

Young people with a mental disorder in the most recent survey were more likely to experience multiple disorders, with greater severity, higher distress, and more days of incapacity due to mental ill health, compared to 2007. These findings reflect not only an increase in prevalence but also in severity and functional impact, indicating that mental disorders are exerting a more substantial effect on multiple aspects of young people’s lives. Overall, mental health conditions experienced by young people were demonstrably more debilitating than in 2007. Comorbidity trends are also particularly important, as they highlight the growing need for integrated rather than disorder-specific clinical pathways for many young people who will experience more than one mental disorder concurrently.

### Comparison with international surveys of youth prevalence

International, population-representative diagnostic surveys of mental disorders typically report overall population prevalence estimates, with few providing age-specific data for individuals aged 16–24 years, limiting direct comparability with the present findings. More recent population-based surveys using CIDI diagnostic criteria to report prevalence estimates separately by age group have been conducted in Saudi Arabia, and in Sao Paulo Brazil reporting lifetime prevalence of all mental disorders to be approximately 40% and 44% among individuals aged 15–30 years^16^ and 18–34 years respectively.^17^ These lifetime rates are comparable to the 36% 12-month prevalence reported in the most recent Australian survey, although it is noted the Saudi Arabian and Brazilian studies examined different age ranges and different contexts. In contrast, a recent cross-national study using similar methods but examining a younger age group (10–17-year-olds) in Kenya, Indonesia, and Vietnam found substantially lower 12-month prevalence rates of 12.1%, 5.5%, and 3.3%, respectively,^18^ which may be reflective of both the narrower, younger age range and particular contexts of these countries. Findings from the Global Burden of Disease Study suggest regional variation within the Western Pacific. While there are indications that adolescent depression rates have risen in countries such as Australia and New Zealand, declining trends have been observed in others, including China, Singapore, and the Philippines.^19^ These prevalence variations by region demonstrate the importance of regular monitoring of youth population prevalence at a national population level to accurately capture country level variation, as well as change over time.

### Implications

While these findings indicate a shifting diagnostic landscape, it is important to acknowledge that young people demonstrate considerable resilience and adaptability. They are navigating rapid societal changes while emerging as leaders and advocates for their own wellbeing and futures. Recognising this strength alongside the burden of mental disorders provides a more balanced perspective and highlights the potential for prevention and intervention strategies that build on existing capacities. Nevertheless, the rising prevalence of mental disorders among Australian youth signals an urgent need for increased funding, targeted policies and practices to support young people’s mental health including the need to act early in the lifespan. The combination of increased prevalence, severity, and complexity suggests that current systems are unlikely to meet future demand without substantial investment. Early intervention and prevention will be critical to curb these growing prevalence rates. Alarmingly, the increases were not confined to mild or transient disorders or distress; mental disorders were more likely to be severe in the most recent survey and associated with substantial impairment. Previous research indicates Australians wait an average of 12 years before seeking treatment for a mental or substance use disorder.^20^ Delayed intervention increases the likelihood that conditions become entrenched, more costly, harder to treat, and associated with additional comorbidities. Mental disorders during adolescence and early adulthood, a critical developmental period, can also disrupt educational attainment, workforce participation, and other key life transitions.

While this study could not determine what is driving the significant changes over time in youth mental health and substance use disorders, due to the cross-sectional nature of the data collected, several hypotheses are considered below. These hypotheses were collaboratively discussed with members of The Matilda Centre Youth Advisory Board, and the findings contextualised and supplemented by the youth lived-experience author (GB) in a youth commentary (https://doi.org/10.1016/j.lanwpc.2026.101926). Four hypotheses were considered: (i) rising prevalence reflects greater symptom reporting among recent cohorts, compared with earlier generations; (ii) the related prevalence inflation hypothesis,^21^ (iii) contemporary young people may be less resilient than previous generations; and (iv) observed increases represent real increases in the incidence of mental disorders among more recent cohorts. These hypotheses are considered and responded to in the accompanying youth commentary (https://doi.org/10.1016/j.lanwpc.2026.101926). Regardless of what is driving these changes, they represent a significant increase in prevalence and may represent the changing landscape of risk and protective factors. These hypotheses require longitudinal and developmental cohort data to evaluate.

### Strengths and limitations

A key strength is the use of the same diagnostic interview (WHO-CIDI) in both surveys, enabling unusually robust temporal comparison. It is noted the interview did not assess lower prevalence disorders (for example, schizophrenia or personality disorders), meaning that reported rates do not capture these lower prevalence conditions and are likely underestimates. The lowest age range of participants was 16 years, meaning that younger adolescents and children were also not represented in the current study. However, it is noted that Australia is undertaking a nationally representative survey of Australian children aged 4–17 years (Young Minds: Our Future survey), which will cover these younger cohorts. Prevalence estimates in the most recent survey were significantly higher than estimates in 2007. As data collection for the 2020–2022 survey coincided with the COVID-19 pandemic, prevalence estimates may have been influenced by this unique context.^22^ Elevated rates of social phobia, marked increases in agoraphobia, and declines in alcohol use disorders observed in the most recent survey could in part reflect pandemic-related conditions. Although the current study could not quantify the magnitude of this effect, evidence indicates that rising mental ill health among young people predated the onset of the pandemic.^23^ A Norwegian population-based study using repeated CIDI assessments before and during the first six months of the pandemic reported stable or even declining rates of mental disorders during the first six months of the COVID-19 pandemic.^24^ Whether this pattern persisted over the longer term remains unknown, and its relevance to the Australian context is uncertain. In contrast, research based on symptom level measures (typically assessing symptoms over shorter periods–e.g., two-weeks) found significantly higher rates of clinically elevated symptoms among children and adolescents during the COVID-19 pandemic, as shown in a global review.^22^ As mentioned above, the mechanisms underlying these changes in prevalence require further exploration. The possibility that these results are explained by compositional shifts across time in various demographic strata would require deeper investigation, possibly through techniques like age-period-cohort analysis, which was beyond the scope of the current study. Lastly, even though the data are weighted to approximate the general population there is still the possibility of residual bias due to differential missingness, particularly missingness that is related to the presence of a mental disorder.

### Conclusions

Mental disorders among Australian young people aged 16–24 years of age continue to rise and have become increasingly complex and impairing since last measured nationally in 2007. Mental disorders are not only becoming more prevalent among youth, but are also more likely to be severe, more likely to co-occur within the same 12-month period, and result in greater impairment. These findings highlight an urgent need for scalable, early, and integrated intervention approaches that respond to a youth mental health burden characterised by higher severity and comorbidity.

## Contributors

LB and TS conceptualized the paper. SJ undertook the statistical analysis under the oversight of MS and TS. LB, SJ and TS drafted the initial article. GB provided youth lived experience review and input into the manuscript. All authors reviewed and approved the final manuscript. MT and TS were members of the technical advisory group for the NSMHWB. SJ, TS & MS had access to the raw data and verified the data. LB and TS had final responsibility for the decision to submit the manuscript.

## Data sharing statement

The detailed microdata used in this study can be obtained by seeking approval from the Australian Bureau of Statistics.

## Declaration of generative AI and AI-assisted technologies in the manuscript preparation process

Microsoft Copilot was used to copyedit the manuscript (e.g., editing written text and to condense wording). After using this tool, the authors reviewed & edited content as needed and take full responsibility for the content of the published article.

## Declaration of interests

LB and LS disclose they receive a salary from the University of Sydney, which is partially funded by a fellowship from NHMRC. TS and SJ discloses they receive a salary from the University of Sydney. LS discloses grants from the Australian Department of Health and Aged Care, Australian National Health and Medical Research Council and royalties for views of an on-demand webinar delivered for the Mental Health Academy summarizing clinical research on the prevention and treatment of co-occurring anxiety and alcohol use disorder. KP’s salary is supported by a NHMRC Clinical Trials and Cohort Study grant. NN & MT are developers of the OurFutures Vaping program, which has been commercialised by the University of Sydney and is being distributed through the not-for-profit organisation, Our Futures Institute. NN & MT are non-executive directors of OurFutures Institute, a joint social enterprise not for profit, established in 2022 to distribute the OurFutures programs and maximise social well-being. JM reports infrastructure support from the University of Sydney and PhD scholarships from Headspace and the Australian Government RTP scheme. MT reports support from NHMRC grant APP1195284. CC reports support from NHMRC grant APP2026552. GB is supported by The Matilda Centre, The University of Sydney and reports receiving a grant from Children and Young People with Disability, as well as acting on the Youth Advisory Board for The Matilda Centre and the following volunteer roles; Board Director of Children and Young People with Disability Australia, National and Queensland Executive of EMILY’s List Australia, Branch Executive of the Australian Labour Party. MS, LG, IK and MT report no COI.
