# Supplementary material for: Rates and impact of mental disorders among Australian youth: prevalence, severity, and trends from two national psychiatric surveys
Source: Lancet Reg Health West Pac. 2026 Jul 23;72:101925. doi: 10.1016/j.lanwpc.2026.101925 (PMC13478814; doi:10.1016/j.lanwpc.2026.101925)
Supplement: Supplementary file 1 [file mmc1.pdf]

Rising rates and impact of mental disorders among Australian youth: Prevalence, severity, and trends from two national psychiatric surveys.

Supplementary materials

Table S1. Prevalence estimates of any past 12-month mental disorder by demographic characteristics in the 2020/2022 and 2007 surveys .... Page 2

Table S2. Interactions between 2007 & 2020/22 demographic variables…………………………………Page 3

Rising rates and impact of mental disorders among Australian youth: Prevalence, severity, and trends from two national psychiatric surveys.

Table S1. Prevalence estimates of any past 12-month mental disorder by demographic characteristics in the 2020/2022 and 2007 surveys

Demographic variable	2020/22 Prevalence	SE	2007 Prevalence	SE
**Employment**				
Employed	36.64%	1.84	24.31%	1.83
Unemployed	45.45%	4.74	28.17%	6.51
Not in the workforce	31.44%	2.61	19.38%	2.36
**Residence**				
Metro	35.93%	1.70	24.27%	1.78
Inner regional	38.89%	3.56	24.68%	2.27
Outer regional	33.49%	5.44	15.26%	2.89
**Birth country**				
Australia	39.33%	1.64	25.70%	1.46
English speaking	35.68%	5.39	20.58%	5.23
Not English speaking	16.82%	2.94	8.82%	2.08
**IRSD**				
IRSD Q1	36.71%	2.47	22.40%	2.13
IRSD Q3-Q4	36.69%	2.10	23.34%	2.60
IRSD Q5	34.62%	2.74	25.02%	2.88

SE: Standard error; IRSD: Index of Relative Socio-economic Advantage and Disadvantage; Q1 represents highest advantage Quartile

Table S2. Interactions between 2007 & 2020/22 demographic variables

		Estimate	SE	t value	Pr (>|t|)	OR	95% CI	95% CI
**Remoteness interaction model**	2020/22 Survey (vs 2007 Survey)	0.559	0.120	4.598	<0.0001	1.75	1.37	2.23
Reference level: 'Major Cities of Australia'	Inner regional	0.022	0.147	0.149	0.881	1.02	0.76	1.37
	Other areas	-0.576	0.261	-2.207	0.029	0.56	0.33	0.94
	2020/22 survey and inner regional	0.104	0.225	0.464	0.643	1.11	0.71	1.73
	2020/22 survey and other areas	0.468	0.374	1.253	0.212	1.60	0.76	3.35
**Country of birth interaction model**	2020/22 Survey (vs 2007 Survey)	0.624	0.102	6.115	<0.0001	1.87	1.53	2.29
Reference level: 'Australia'	Main English-speaking countries	-0.289	0.313	-0.924	0.358	0.75	0.40	1.39
	Other countries	-1.275	0.271	-4.698	<0.0001	0.28	0.16	0.48
	2020/22 and Main English-speaking	0.136	0.400	0.341	0.734	1.15	0.52	2.52
	Year2020/22 and other countries	0.113	0.355	0.319	0.750	1.12	0.55	2.26
**Tobacco smoking interaction model**	2020/22 Survey (vs 2007 Survey)	0.826	0.108	7.625	<0.0001	2.84	1.84	2.83
Reference level: 'Not a current tobacco smoker'	Tobacco smoker	1.129	0.164	6.899	<0.0001	3.09	2.24	4.28
	2020/22 Survey and tobacco smoker	-0.145	0.280	-0.517	0.517	0.87	0.50	1.51
**IRSD interaction model**	2020/22 Survey (vs 2007 Survey)	0.688	0.161	4.262	<0.0001	1.99	1.45	2.74

Reference level: 'IRSD Q1'	IRSD Q3-Q4	0.053	0.206	0.256	0.798	1.05	0.70	1.59
	IRSD Q5	0.145	0.189	0.765	0.446	1.16	0.79	1.68
	2020/22 survey and Q3-Q4	-0.044	0.247	-0.178	0.859	0.96	0.59	1.56
	2020/22 survey and Q5	-0.226	0.245	-0.923	0.358	0.80	0.49	1.30
**Employment interaction model**	2020/22 Survey	0.583	0.126	4.616	<0.0001	1.79	1.40	2.30
Reference level: 'Currently employed'	Unemployed	0.199	0.348	0.574	0.567	1.22	0.61	2.43
	Not in the workforce	-0.290	0.190	-1.526	0.130	0.75	0.51	1.09
	2020/22 Survey and unemployed	0.170	0.407	0.418	0.677	1.19	0.53	2.66
	2020/22 survey and not in the workforce	0.063	0.243	0.258	0.797	1.06	0.66	1.72

SE: Standard error; Odds ratio; 95% CI: 95% confidence interval; Pr (>|t|): Covariate p-value, IRSD: Index of Relative Socio-economic Advantage and Disadvantage; Q1 represents highest advantage Quartile
